# Presence, Co-Occurrence, and Daily Intake Estimates of Aflatoxins and Fumonisins in Maize Consumed in Food-Insecure Regions of Western Honduras

**DOI:** 10.3390/toxins15090559

**Published:** 2023-09-07

**Authors:** Luis Sabillón, Jackeline Alvarado, Alejandra Leiva, Rodrigo Mendoza, Raúl Espinal, John F. Leslie, Andréia Bianchini

**Affiliations:** 1 Department of Family and Consumer Sciences, New Mexico State University, Las Cruces, NM 88003, USA; 2 Center of Excellence in Sustainable Food and Agricultural Systems, New Mexico State University, Las Cruces, NM 88003, USA; 3 Department of Food Science and Technology, Zamorano University, San Antonio de Oriente P.O. Box 93, Honduras; 4 Department of Food Science and Technology, University of Nebraska-Lincoln, Lincoln, NE 68588, USA; 5 The Food Processing Center, University of Nebraska-Lincoln, Lincoln, NE 68588, USA; 6 Department of Plant Pathology, Kansas State University, Manhattan, KS 66506, USA

**Keywords:** mycotoxins, exposure, food safety, public health, smallholder farmers, subsistence farming

## Abstract

Foodborne mycotoxins are a significant food safety risk in developing countries. Our objective was to determine the occurrence of and exposure levels to aflatoxins (AFs) and fumonisins (FBs) in maize intended for human and animal consumption in food-insecure regions of western Honduras. Total AFs and FBs were quantified with a monoclonal antibody-based affinity spectrofluorimetric method. FBs were detected in 614/631 samples of maize destined for human consumption at 0.3 to 41 mg/kg (mean, 2.7 mg/kg). Of the 614 positive samples, 147 had FB levels exceeding the U.S. Food and Drug Administration (FDA) advisory threshold of 4.0 mg/kg. AFs were detected in 109/631 samples of maize for human consumption with concentrations between 1.0 and 490 µg/kg (mean, 10 µg/kg). AF levels in 34 samples exceeded the FDA regulatory limit (i.e., 20 µg/kg). The average probable daily intake of AFs in western Honduras ranged from 0 to 260 ng/kg body weight/day, and for FBs, the average probable daily intake ranged from 17 to 53 μg/kg body weight/day. AFs and FBs co-occurred in 106/631 samples with 60 samples containing both toxins at levels greater than the FDA regulatory levels. Samples of maize intended for animal feed had significantly higher AF (mean, 22 µg/kg) and FB (mean, 7.6 mg/kg) contamination levels than those observed in samples destined for human consumption. Thus, the maize supply chain in western Honduras is contaminated with mycotoxins at levels that pose health risks to both humans and livestock. More effective mycotoxin surveillance and implementation of effective mitigation strategies are needed to reduce mycotoxin contamination and exposure.

## 1. Introduction

Maize (*Zea mays* L.), one of the world’s most important staple grain crops, is susceptible to contamination by multiple mycotoxins, which are toxic secondary metabolites produced by some filamentous fungi. Aflatoxins and fumonisins are among the mycotoxins of public health and agroeconomic significance afflicting maize crops worldwide. Exposure to these mycotoxins through the diet can cause a variety of adverse health effects and pose a serious health threat to both humans and domesticated animals [1].

Aflatoxins (AFs) are among the most poisonous mycotoxins and are mainly synthesized by *Aspergillus flavus* and *Aspergillus parasiticus* [2]. These fungi usually invade the grain during storage; however, they may thrive in the field in dry weather conditions. The most common types of aflatoxins (AFs) are aflatoxin B_1_ (AFB1), B_2_ (AFB2), G_1_ (AFG1), and G_2_ (AFG2). AFB1 is considered the most potent naturally occurring mutagenic and carcinogenic mycotoxin [3]. Acute exposure to high levels of Afs can be fatal, while chronic exposure has been associated with impaired growth in children, immunosuppression, and liver cancer around the world [4,5,6].

Fumonisins (FBs) are produced by several species of the genus *Fusarium*, especially *F. verticillioides* and *F. proliferatum* [2]. *Fusarium* spp. are generally regarded as soil-borne fungi, and numerous species are important plant pathogens with a worldwide distribution [7]. Fumonisin B_1_ (FB1), B_2_ (FB2), and B_3_ (FB3) are the most common *Fusarium* mycotoxins associated with maize. These toxins, in particular FB1, have been related to growth stunting and esophageal cancer in humans [8,9] and to liver, kidney, and neurotoxicity in animals [10].

Different classes of mycotoxins can co-occur in a single agricultural commodity. The effects of interactions of multiple mycotoxins on human health are not yet fully understood; however, some research studies highlight that co-exposure to AFs and FBs may exacerbate child growth impairment and lead to an increase in chronic liver disease in humans [11,12]. To limit the exposure and toxic effects of mycotoxins in humans, regulatory agencies have established maximum tolerated levels. For instance, in maize intended for human consumption, the U.S. Food and Drug Administration set a regulatory limit for AFs (20 µg/kg (20 ppb); [13]) and an advisory level for FBs at (4 mg/kg (4 ppm); [14]). Likewise, the European Commission has established maximum limits for AFs (4 ppb) and FBs (1 ppm) contamination in maize destined for direct human consumption [15]. In addition, based on available toxicity data, the Joint FAO/WHO Expert Committee on Food Additives (JECFA) established a provisional maximum tolerable daily intake (PMTDI) of 2 μg/kg bw/day for FB1, FB2, and FB3, alone or in combination [16]. While there is no official PMTDI for AFs, previous studies have recommended that daily exposure levels should not exceed 1 ng/kg bw/day [16,17].

In the Republic of Honduras, maize is the leading crop and the main dietary staple for the country’s overwhelmingly rural and indigenous population. The western region of the country is located within the so-called “Dry Corridor”, which is a climate-fragile and impoverished stretch of land on Central America’s Pacific coast. Much of the maize produced in western Honduras is cultivated, harvested, and handled via subsistence-oriented agricultural practices, which are strongly connected to the Mayan-Lenca heritage of many local residents. Factors such as adverse climatic conditions, inadequate agricultural practices, and poor handling and storage of maize crops associated with subsistence farming may increase exposure to life-threatening fungal toxins [18,19].

Information about the levels of mycotoxin contamination in Honduran maize produced and consumed in rural communities is almost non-existent. However, mycotoxin surveillance studies carried out in the neighboring Republic of Guatemala suggest that the population living in the dry corridor region may be exposed to AF and FB contamination levels in maize that far exceed the United States and European Union import limits [12,20,21]. Furthermore, follow-up research studies in this Guatemalan region found an association between mycotoxin dietary intake and the incidence of child growth impairment and chronic liver disease [12,22,23].

Fueled by prolonged droughts, highly erratic weather patterns, and high socioeconomic vulnerability, Honduras’s dry corridor region is home to systemic hunger and malnutrition. In a recent survey conducted by the International Food Policy Research Institute [24], 25 percent of children living in this rural region of Honduras suffer from stunting. Maize constitutes a dominant part of the diet in this region, but the magnitude of contamination by and exposure to AFs and FBs is unknown. The objectives of this study were (i) to determine the occurrence and co-occurrence of AFs and FBs along the maize value chain in western Honduras and (ii) to estimate the daily intake of these mycotoxins through the maize being consumed by adult individuals across the region. Our working hypothesis was that, given the climatic conditions and agricultural practices in this region, the prevalence of AFs and FBs in the maize supply chain is high, and humans and animals consuming this grain would be exposed to levels of these toxins that far exceed the known safe exposure limits. Our results advance the field by confirming results from Guatemala and extending them to another country, for which we provide the first firm data. These data are essential for the development of management and remediation strategies to reduce the health and economic risks posed by these toxins in western Honduras.

## 2. Results

### 2.1. Aflatoxin Contamination Levels and Dietary Exposure

The presence of aflatoxin in maize intended for human consumption in western Honduras was relatively low (Table 1). In general, most samples (*n* = 522/631 [83%]) had no measurable amounts of AFs. However, aflatoxin contamination levels exceeding the US regulatory limit (i.e., 20 µg/kg) were found in 25/506 samples from rural areas and 9/125 samples from urban areas. If the stricter European limit (i.e., 4 μg/kg) is used, then 58 of the rural samples and 21 of the urban samples exceeded the limits. If detectable contamination levels (i.e., ≥1 µg/kg) were considered, then 16% of the rural samples and 23% of the urban samples were contaminated (Table 1). The average AF concentration in rural and urban samples that did not meet FDA guidelines was 116 and 84 µg/kg, respectively; while those samples that exceeded EU limits had an average concentration of 56 µg/kg in rural areas and 42 µg/kg in urban areas. Overall, differences in AF prevalence and the average concentration between samples from urban and rural areas were not significant (*p*  >  0.05).

**Figure 1:**
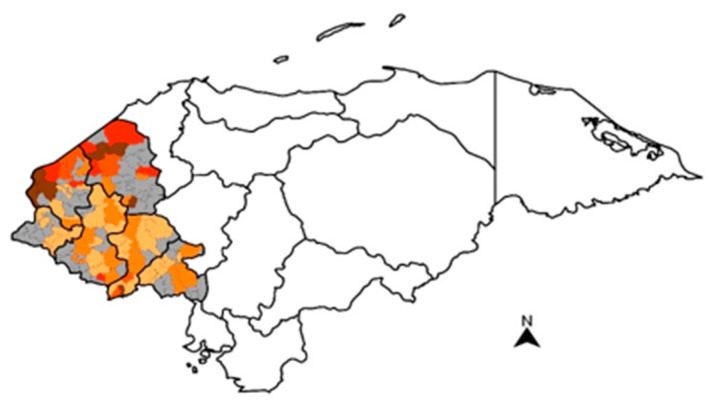
Heatmap based on average aflatoxin concentrations in maize samples collected in western Honduras in 2017–2018. The intensity of color for each municipality corresponds to the related total aflatoxin level. Range of contamination: <1 μg/kg (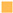), ≥1–4 μg/kg (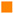), >4–10 μg/kg (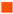), >10–20 μg/kg (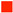), >20 μg/kg (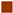). Non-analyzed municipalities are shown in gray (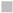).

AF contamination in maize, while present, was not widespread in the region of study (Figure 1). Detectable levels of AFs (i.e., >1 μg /kg) were more frequent in maize from the departments of Santa Bárbara, with a prevalence of 34% and 41% in rural and urban samples, respectively (Table 1). When samples from both rural and urban areas are combined, 36% of total samples (*n* = 43/121) for human consumption collected in Santa Bárbara had measurable levels of AFs, with 19/121 above the FDA maximum and 35/121 above the EU maximum.

Average probable daily intakes (PDI) of AFs in the region of study, through the consumption of contaminated maize, were considerably higher than the recommended exposure limit of 1 ng/kg bw/day for both genders. For instance, in Santa Bárbara, adult individuals living in rural areas were exposed to average PDIs of 140 to 180 ng/kg bw/day; while those living in urban settings had higher intake levels ranging between 210 and 260 ng/kg bw/day (Table 2). In general, women and men living in rural and urban areas of western Honduras could be exposed to maximum intake levels of AFs that are 50 to 100 times higher than tolerable daily intakes.

Samples of maize destined for animal feed also were collected in rural communities. Through visual inspection, smallholder farmers separate diseased and/or damaged kernels and then use the poor-quality grain to feed their farm animals. In general, these samples showed higher AF prevalence and concentrations than those found in those destined for human consumption (Table 1). From the total number of samples, 34% (*n* = 37/109) exhibited AF levels above the detection limit of the method (i.e., 1 µg/kg), of which 46% (17 samples) had concentrations that surpassed the FDA and EU permissible levels (i.e., 20 µg/kg) in feed materials [15,25].

### 2.2. Fumonisin Contamination Levels and Dietary Exposure

Unlike AFs, FBs were substantially more prevalent and widespread in the maize supply chain of western Honduras. FBs were detected in 614 of 631 samples (97% prevalence) of maize destined for human consumption. Contamination levels ranged from 0.25 to 41 mg/kg (mean, 2.7 mg/kg) in samples from rural areas, and from 0.25 to 16 mg/kg (mean, 3.2 mg/kg) in urban locations (Table 3). Among the positive samples, 73% (*n* = 449/614) were contaminated above the regulatory limit of 1.0 mg/kg set by the European Commission, while 24% (*n* = 147/614) had levels of FBs above the FDA advisory threshold of 4.0 mg/kg. The difference in the average levels of FBs between samples from urban and rural areas was not significant (*p*  >  0.05).

These findings are consistent with those of Julian et al. [26] who found that maize samples often tested negative for AFs in eastern Honduras, while most were highly contaminated with FB1, ranging from 0.1 to 6.6 mg/kg. In the neighboring Republic of Guatemala, a survey by Torres et al. [21] reported a high incidence of FB contamination (88.6%, *n* = 209/236) in maize samples collected from local markets, with an average concentration of 3.6 mg/kg. More recently, Mendoza et al. [20] found FB contamination, ranging from 0.4 to 31 mg/kg, in maize samples collected from 13 (52%, *n* = 25) smallholder farms in Huehuetenango, Guatemala.

**Figure 2:**
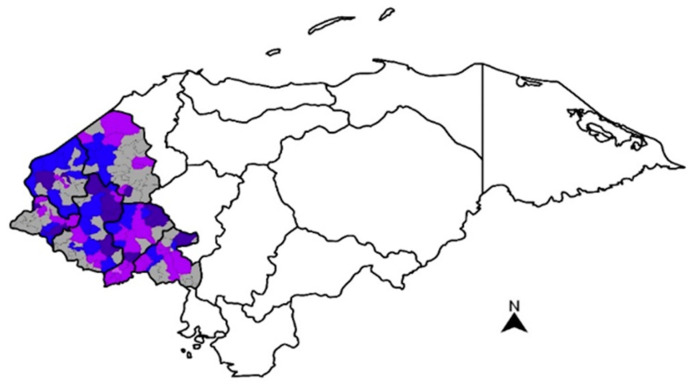
Heatmap based on average fumonisin concentrations in maize samples collected in western Honduras in 2017–2018. The intensity of color for each municipality corresponds to the related total fumonisin level. Range of contamination: <0.25 mg/kg (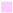), ≥0.25–1 mg/kg (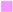), >1–3 mg/kg (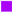), >3–4 mg/kg (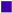), >4 mg/kg (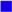). Non-analyzed municipalities are shown in gray (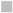).

FBs were a substantial contaminant of the maize supply chain in western Honduras, as levels considered a public health concern were often found throughout the region (Figure 2). The maize samples most highly contaminated with FBs were obtained from local markets in the department of La Paz, with an average concentration level of 5.4 mg/kg (Table 3).

To limit the exposure and toxic effects of FBs, JECFA established a provisional maximum tolerable daily intake (PMTDI) of 2.0 μg/kg bw/day for FB1, FB2, and FB3, separately or combined [16]. In the current study, estimated exposure levels across the region surpassed the PMTDI of 2.0 μg/kg bw/day for both genders (Table 4). Overall, on average, FB exposure was 14 (29 μg/kg bw/day) and 12 (24 μg/kg bw/day) times higher than the PMTDI for women and men, respectively. When maximum contamination levels were considered, for example in La Paz, FB exposure in adult individuals exceeded the PMTDI by up to 39 times (78 μg/kg bw/day).

Maize from samples intended for animal feed had significantly higher FB contamination levels (mean, 7.6 mg/kg) than those observed in maize destined for human consumption in rural (mean, 2.7 mg/kg) and urban areas (mean, 3.2 mg/kg) (Table 3). The highest FB contamination levels in samples destined for animal consumption were found in the departments of Copán and Lempira, with an average concentration of 9.9 and 9.7 mg/kg, respectively. From the total number of samples, 55% (*n* = 57/109) had concentrations of FBS that surpassed the FDA and EU permissible levels (5 mg/kg) in feedstuff for equids and swine [14,15].

### 2.3. Co-Occurrence of Aflatoxin with Fumonisin

AFs and FBs were found simultaneously in 15% (*n* = 77/506) and 23% (*n* = 29/125) of samples of maize destined for human consumption in rural and urban areas, respectively. Thirty-four (32%) of the 106 samples that contained both AFs and FBs had levels of aflatoxin above the FDA maximum regulatory limit of 20 µg/k,; while 26 (25%) samples exceeded the fumonisin advisory threshold of 4.0 mg/kg. When EU regulations are considered, 83/106 and 79/106 samples had FBs and AFs, respectively, above the maximum permitted levels. The levels of AFs and FBs in these samples ranged from 1.0 to 490 µg/kg (mean, 39 µg/kg) and from 0.3 to 16 mg/kg (mean, 3.1 mg/kg), respectively.

In the case of maize destined for animal feed, AFs and FBs were found together in 35 (32%) of 109 samples. Of the 35 samples that contained both toxins, 17 (49%) and 23 (66%) samples contained levels of AFs and FBs, respectively, considered unsafe for many animal species. The average aflatoxin contamination level in animal feed samples that contained both toxins was 67 µg/kg, while fumonisin was present at a mean concentration of 7.5 mg/kg.

## 3. Discussion

AFs are among the most important genotoxic carcinogens in foods for which there is no fixed tolerable daily intake (PMTDI); however, JECFA has recommended that daily exposure levels should not exceed 1 ng AFs/kg bw, as it may induce liver cancer [16]. In general, the average AF exposure estimates in the region of study were 57 and 45 times higher than the PMTDI suggested by JECFA for women and men, respectively. Higher-than-recommended AF intake levels were also reported in a mycotoxin exposure assessment conducted by Mendoza et al. [20] in maize crops produced by subsistence farmers in the western highlands of the neighboring Republic of Guatemala, with estimated daily intakes varying from 10 to 850 ng AFs/kg bw.

The results obtained in the present study also indicated that, regardless of the source of maize, populations living in western Honduras are at risk of exposure to harmful levels of fumonisins. In general, FB exposure was 14 and 12 times higher than the PMTDI of 2 μg/kg bw/day established by JECFA for women and men, respectively. Similar findings were reported in Guatemala, where an FB exposure assessment revealed that women living in urban areas may be exposed to an average daily intake of 3.5 μg FBs/kg bw/day when consuming nixtamalized maize products, while in rural locations, the exposure averaged 16 μg FBs/kg bw/day [21]. In worst-case scenarios, FB exposure levels in rural Guatemala reached 69 μg/kg bw/day, 35 times the established PMTDI. More recently, Mendoza et al. [20] reported that rural communities in the western highlands of Guatemala are also exposed to a considerably high FB intake (2.9 to 310 μg /kg bw/day) through the consumption of maize-based products.

The high incidence of FB contamination in maize suggests that fumonisin-producing fungi, such as *F. verticillioides* and *F. proliferatum*, are likely to be common and widely disseminated plant pathogens in the region of study. In fact, previous mycological surveys carried out in Honduras between 1992 and 1995 showed that *Fusarium* spp. were the predominant fungal isolates in maize samples from field and storage, while the incidence of *Aspergillus* spp. was very low [26,27]. In western Honduras, a warm and humid climate prevails, which is generally conducive to the proliferation of fumonisin-producing *Fusarium* species in the field [21]. This climate could explain both the high incidence of *Fusarium* infection in maize crops reported by previous research studies in the region and the common occurrence of fumonisin contamination observed in the present study. In fact, climatic conditions represent a key factor in driving the composition of fungal communities and mycotoxin production in staple crops at pre- and post-harvest stages [28]. Climate change and extreme weather events are expected to exacerbate the levels of fungal infection and mycotoxin contamination in staple cereals [29,30].

In western Honduras, livestock farming is an integral component of smallholder livelihoods. Beef and dairy cattle, swine, and poultry are among the most popular animal species raised in the region through traditional farming methods for food and other necessities. In the present study, the AF and FB contamination levels observed in maize destined to feed these farm animals may cause a wide range of adverse effects that would reduce their performance (Table 1 and Table 3). Chronic dietary exposure to AFs affects animal health not only by increasing morbidity and mortality, but also impacts productivity by reducing growth, reproductive capacity, and the production of eggs and milk [31]. Furthermore, the biotransformation of ingested AFs could lead to the deposition and accumulation of toxic metabolites in animal-derived foods, including edible tissues, milk, and eggs, thus potentially increasing human exposure to mycotoxins [32,33]. Similarly, FB dietary intake has been linked to species-specific diseases including leukoencephalomalacia in horses, pulmonary edema in pigs, and liver and kidney toxicities in horses, cattle, pigs, chickens, sheep, and rodents [10,34,35].

AFs and FBs, separately, have been linked to multiple adverse health effects in both humans and animals. However, when co-exposure occurs, in vivo and in vitro studies suggest that these mycotoxins may act synergistically or additively to enhance hepatotoxicity and hepatocarcinogenic effects. For instance, Gelderblom et al. [36] showed that AFB1 exposure enhanced the FB1 carcinogenic effect in rat livers, whereas McKean et al. [37] reported that FB1 increased the acute toxicity of AFB1 in F344 rats and mosquito fish. In humans, numerous epidemiological studies have shown that co-exposure to these mycotoxins may lead to impaired growth in children and an increase in chronic liver disease [11,12,38]. The natural co-occurrence of AFs and FBs in maize has been reported in several countries, including Nigeria [39], Tanzania [40], Ghana [41], Guatemala [12], and Zimbabwe [42]. The high incidence of FB contamination observed in this study suggests that *Fusarium* species are endemic in the region. In addition to fumonisins, other *Fusarium* spp. also produce additional important mycotoxins, including trichothecenes (e.g., deoxynivalenol, nivalenol, T-2 toxin) and zearalenone. Consequently, co-exposure to multiple mycotoxins is likely to occur in western Honduras, where maize-based foods are a dietary staple. A comprehensive mycological study in the “Dry Corridor Region” is warranted to better understand the geographic distribution and mycotoxin-production potential of endemic plant-pathogenic fungi, such as *Fusarium* and *Aspergillus* species.

In conclusion, the present study provides evidence that fumonisin is a prevalent, widespread contaminant of maize for human consumption and animal feed in western Honduras. Estimated exposure levels for adult individuals across the region surpassed the PMTDI set by WHO-JECFA for both toxins. The presence of mycotoxins in maize-based foods is likely to remain a food safety challenge in western Honduras as the erratic weather patterns that prevail in the region are conducive to fungal growth and mycotoxin production. Thus, there is an urgent need for the continuous monitoring of AFs and FBs, as well as other fungal metabolites associated with this vulnerable grain commodity. Establishing and enforcing regulatory limits for these mycotoxins throughout the maize value chain in Honduras is warranted in order to reduce exposure and improve public health. Additionally, it is important to raise awareness among smallholder farmers, traders, and consumers in the region regarding the risk associated with mycotoxins, while equipping them with effective mitigation strategies to prevent and reduce exposure.

## 4. Materials and Methods

### 4.1. Study Area and Sampling Methodology

**Figure 3:**
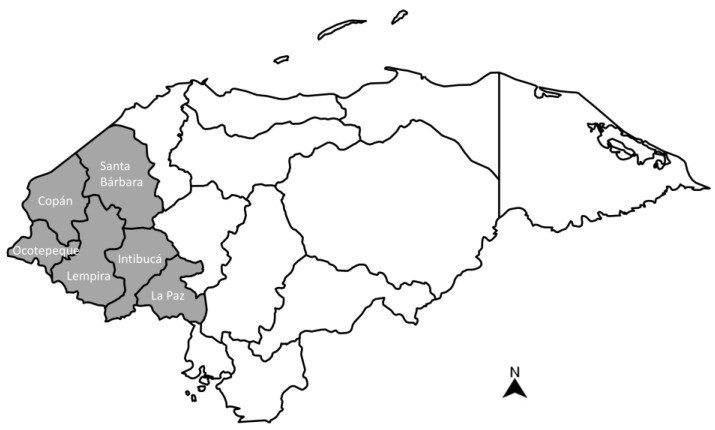
Map of the Republic of Honduras showing the region (grey-colored area) where maize samples were collected. The name of each department (i.e., Copán, Santa Bárbara, Intibucá, La Paz, Lempira, and Ocotepeque) appears in Spanish.

The Republic of Honduras is administratively divided into 18 departments. The study was conducted in the western part of the country (Figure 3), which comprises six departments (i.e., Copán, Santa Bárbara, Intibucá, La Paz, Lempira, and Ocotepeque). This geographic area falls within the “Dry Corridor”, a region in Central America characterized by extreme weather events, food insecurity, and a high prevalence of chronic malnutrition. This vulnerable region in Western Honduras is designated as a Feed the Future (FTF) Zone of Influence (ZOI) [43]. FTF is the U.S. government’s global hunger and food security initiative that supports country-driven approaches to address the root causes of poverty, hunger, and undernutrition.

Each department is further divided into administrative divisions called municipalities. Several municipalities within these six departments were selected for sample collection based on population density and population-based indicators developed by FTF to monitor the impact of projects conducted in this region. These indicators include poverty (i.e., people living on < US$1.25/day), the proportion of underweight nonpregnant women between the ages of 15 and 49, and indicators associated with underdevelopment in children < 5 years of age, including stunting, wasting, and underweight [24]. Stunting in children < 5 age was weighted 3× more heavily than the other sample selection criteria. Therefore, the samples we evaluated, while being a good representation of the study area, favor municipalities where stunting in children is high.

For each department, only municipalities with the worst indicators (e.g., the highest prevalence of stunting in children) were incorporated into the study. Samples included maize destined both for human consumption (631 samples) and for animal feed (109 samples). Sample origin (740 total samples) was distributed among departments and selected municipalities, based on population density and the criteria described above. Samples assigned to each municipality were subdivided into those with rural and urban community origins. In rural communities, samples were arbitrarily collected from smallholder subsistence farmers who planted and harvested their own maize crops and who were located in an area that was accessible by road. For urban locations, samples were obtained from wholesale markets and retail stores. Samples of maize considered low quality, and destined for animal feed, were collected only from farmers in rural communities.

### 4.2. Collection and Storage of Maize Samples

All 740 maize samples were collected between November 2017 and October 2018. Of the 631 samples intended for human consumption, 506 originated in rural areas and 125 in urban areas, and the 109 samples destined for animal feed all originated in rural areas. Samples could be freshly harvested maize (<3 days of storage) (110 samples) and maize that had been stored for different lengths of time, ranging from 20 days to 1 year (630 samples). Sample storage methods prior to collection varied and included both conventional (e.g., bags, metal silos, and plastic drums) and traditional methods (e.g., trojas and tabancos).

When samples were collected, several 0.5-kg sub-samples were taken from storage containers with a 1-m long open-handle spiral probe (Seedburo Equipment, Des Plaines, IL, USA). Sub-samples were thoroughly mixed, and 2.2 kg of the composite sample was collected, labeled, and placed in a double polyethylene bag for storage and subsequent testing. On the same day of collection, samples were transported to a centralized location in each department where they were stored at −20 °C until being transferred to the Food Analysis Laboratory at Zamorano University for mycotoxin analysis. In the laboratory, samples were kept at −20 °C until the analysis was complete. The unused sample material, after mycotoxin analysis, was disposed of by burying it in an earthen pit with dimensions that tripled the material volume.

### 4.3. Sample Preparation for Mycotoxin Analysis

All maize samples were ground with a Romer Series II laboratory mill (Romer Labs Inc., Newark, DE, USA) to pass through a 20-mesh sieve. The mill was cleaned according to the manufacturer’s instructions to avoid cross-contamination between samples. Before milling, moisture content was measured with a DICKEY-john GAC 500XT (DICKEY-john, Auburn, IL, USA). If the moisture content was >15%, then the grain was dried in a forced-air oven at 40 °C until the moisture content was between 13 and 15%. The ground material was collected in a new polyethylene bag, thoroughly mixed, and subsequently analyzed for AFs and FBs using a monoclonal antibody-based affinity spectrofluorimetric system (VICAM, Milford, MA, USA).

### 4.4. Mycotoxin Analysis

All chemicals and solvents (e.g., sodium chloride, methanol, phosphate-buffered saline, and tween), immunoaffinity columns, mycotoxin calibration standards, laboratory supplies (e.g., disposable plastic beakers, filter paper, and disposable cuvettes), and equipment (e.g., Series-4EX Fluorometer) used in this study for mycotoxin analysis were obtained from VICAM (Milford, MA, USA). All reagents used in this study were analytical grade, unless otherwise specified.

#### 4.4.1. Extraction and Quantification of Aflatoxins

To measure the levels of total AFs (i.e., AFB1, AFB2, AFG1, and AFG2), ground samples (25 g) were blended with 5 g of sodium chloride and 125 mL of 70:30 (*v*/*v*) methanol: water in a Waring blender jar at high speed for 2 min. The mixture was filtered through 24 cm VICAM fluted filter paper. Fifteen milliliters of the filtered extract were diluted with 30 mL of deionized water and re-filtered through 1.5 µm glass microfiber filter paper into a clean vessel. The filtrate (15 mL) was then passed through an AflaTest^®^ immunoaffinity column (VICAM) at a flow rate of approximately 1 drop/second followed by two 10-mL washing cycles with deionized water at 1–2 drops/second. AFs were eluted from the column with 1 mL of HPLC-grade methanol at a rate of 1 drop/second into a glass cuvette. The eluate was mixed with 1 mL of freshly made AflaTest^®^ Developer solution and the fluorescence of the mixture was measured in a pre-calibrated fluorometer (VICAM Series-4EX Fluorometer). The limit of detection (LOD) and the quantification range were 1.0 µg/kg and 0–50 µg/kg, respectively. For those samples that exceeded the range of quantification, samples were re-analyzed by further diluting the filtered extract with deionized water such that the AF concentration in the diluted sample was <50 µg/kg.

#### 4.4.2. Extraction and Quantification of Fumonisins

To quantify the level of FBs (i.e., FB1, FB2, and FB3), ground samples (50 g) were mixed with 5 g of sodium chloride and 100 mL of methanol:water (80:20 *v*/*v*) in a Waring blender jar. The mixtures were blended at high speed for 1 min and filtered through a 24 cm VICAM fluted filter paper. The extract (10 mL) was diluted with 40 mL of 0.1% Tween-20/2.5% PEG/PBS wash buffer and re-filtered through a 1.5 µm glass microfiber filter paper into a clean vessel. The filtrate (5 mL) was passed through a FumoniTest^®^ immunoaffinity column (VICAM) at a flow rate of approximately 1 drop/second. The column was washed with 6 mL of 0.1% Tween-20/2.5% PEG/PBS wash buffer followed by two 6 mL washing cycles with PBS. FBs were eluted from the column using 1 mL of HPLC-grade methanol at a rate of 1 drop/second and collected in a cuvette. A mixture (1 mL) of freshly made FumoniTest^®^ Developer A and Developer B was added to the eluate and the fluorescence of the mixture was measured in a pre-calibrated fluorometer (VICAM Series-4EX Fluorometer). The LOD and quantification range were 0.25 mg/kg and 0–10 mg/kg, respectively. Those samples that exceeded the range of quantification were re-analyzed after diluting the filtered extract with 0.1% Tween-20/2.5% PEG/PBS wash buffer until the FB concentration in the diluted sample was < 10mg/kg.

### 4.5. Assessment of Dietary Exposure

The dietary exposure to AFs and FBs through maize consumption was estimated for both adult women and men. Recent data on maize dietary intake by Hondurans is not readily available, so other data from Central America were used instead. An average adult male in Central America and Mexico weighs 76.2 kg [44] and consumes approximately 600 g of maize per day [45]. An average adult female in Honduras weighs 58.1 kg [44] and consumes an average of 567 g of maize per day [46].

Dietary exposure to both AFs and FBs was calculated according to Equation (1) and reported as mycotoxin intake in μg or ng/kg of body weight (bw)/day:

(1) $$PDI=(MCL[µg/kg]\times DMC[kg/day])/(BW\left[kg\right])$$

where PDI represents the probable daily intake; MCL signifies the mycotoxin contamination level based on minimum (10th percentile), average, or maximum (90th percentile) concentration of AFs and FBs in samples from the relevant department; DMC denotes the average daily consumption of maize by women or men; and BW represents the average bodyweight for women or men.

Estimated probable daily intakes of AFs and FBs were compared to the provisional maximum tolerable daily intake (PMTDI) of 2 μg/kg bw/day set for fumonisins and to the recommended exposure limit of 1 ng/kg bw/day for AFs [16]. Exposure levels exceeding these limits were considered to be a public health concern.

### 4.6. Data Analysis

Data were analyzed with SAS version 9.3 (SAS Institute, Cary, NC, USA) by using a one-way analysis of variance (ANOVA) to compare changes in aflatoxin and fumonisin contamination in response to sample source/type. ANOVAs were performed by using the GLIMMIX procedure in SAS. Tukey’s multiple comparison test was used to determine significant differences in the incidence and mean of total aflatoxin and fumonisin among samples. All statistical analyses were performed with a significance level of *p* ≤ 0.05. All values below the LOD were treated as “not detected” and assigned values of “zero” for calculating the incidences and means.

## Figures and Tables

**Table 1:** Total aflatoxin contamination levels in maize grain destined for human consumption and for animal feed in western Honduras.

Sample Source (Type) ^a^	Department Name	Number of Samples						Prevalence (%) ^b^	Frequency Distribution (µg/kg)			
		Total	Concentration Range (µg/kg)						Mean ± SE ^c^	Median	10th Percentile	90th Percentile
			x < LOD	LOD ≤ x < 4	4 ≤ x < 10	10 ≤ x < 20	x ≥ 20					
Producer (Human consumption)	Copán	111	93	4	4	4	6	16	11.0 ± 5.5 ^z^	0.0	0	8.0
	Intibucá	80	70	2	1	3	4	13	2.7 ± 1.2 ^z^	0.0	0	3.1
	Lempira	103	91	6	4	2	0	12	0.7 ± 0.2 ^z^	0.0	0	1.1
	La Paz	64	61	0	0	1	2	5	0.9 ± 0.5 ^z^	0.0	0	0
	Ocotepeque	54	49	3	1	1	0	9	0.6 ± 0.3 ^z^	0.0	0	0
	Santa Bárbara	94	62	7	7	5	13	34	18.2 ± 6.9 ^z^	0.0	0	29
	Overall ^d^	506	426 (84%)	22 (4%)	17 (4%)	16 (3%)	25 (5%)	16 ^w^	$\bar{x}$ ^c^ = 6.5 ± 1.8 ^z^			
Market (Human consumption)	Copán	43	34	2	5	0	2	21	2.3 ± 1.0 ^z^	0.0	0	6.7
	Intibucá	16	14	0	1	1	0	13	1.7 ± 1.3 ^z^	0.0	0	3.5
	Lempira	17	13	4	0	0	0	24	0.4 ± 0.2 ^z^	0.0	0	1.7
	La Paz	14	11	1	1	0	1	21	3.6 ± 2.9 ^z^	0.0	0	6.4
	Ocotepeque	8	8	0	0	0	0	0	0 ^z^	0.0	0	0
	Santa Bárbara	27	16	1	2	2	6	41	26.6 ± 15 ^z^	0.0	0	52
	Overall	125	96 (77%)	8 (6%)	9 (7%)	3 (3%)	9 (7%)	23 ^w^	$\bar{x}$ = 7.2 ± 3.3 ^z^			
Producer (Animal feed)	Copán	30	22	2	3	0	3	27	8.8 ± 5.2 ^z^	0.0	0	9.2
	Intibucá	6	5	0	0	0	1	17	81.6 ± 82 ^z^	0.0	0	245
	Lempira	27	17	4	3	1	2	37	3.2 ± 1.3 ^z^	0.0	0	12
	La Paz	11	9	2	0	0	0	18	0.3 ± 0.2 ^z^	0.0	0	1.4
	Ocotepeque	6	4	2	0	0	0	33	0.5 ± 0.3 ^z^	0.0	0	1.4
	Santa Bárbara	29	15	1	0	2	11	48	51.9 ± 20 ^z^	0.0	0	220
	Overall	109	72 (66%)	11 (10%)	6 (5%)	3 (3%)	17 (16%)	34 ^v^	$\bar{x}$ = 22 ± 7.2 ^y^			

^a^ Producer, samples collected from smallholder farmers in rural areas; Market, samples collected from retail stores in urban areas. ^b^ Prevalence was defined as the number of samples with aflatoxin concentration exceeding the limit of detection (LOD) of 1 µg/kg. ^c^ Average concentration of aflatoxin in total samples ± standard error. Average aflatoxin values among departments, within the same sample source, that share the same superscript letter (z) are not significantly different from one another (*p* > 0.05). ^d^ Overall prevalence (v, w) and concentration (x, y, z) values with the same superscript letter do not differ significantly (*p* > 0.05).

**Table 2:** Dietary exposure to aflatoxins in maize grain consumed by households in rural and urban areas of western Honduras.

Sample Source ^a^	Department Name	Women (ng/kg bw/day) ^b^			Men (ng/kg bw/day) ^b^		
		10th Percentile ^c^	Average ^d^	90th Percentile ^e^	10th Percentile ^c^	Average ^d^	90th Percentile ^e^
Rural Areas (Producer)	Copán	0	110	80	0	90	60
	Intibucá	0	30	30	0	20	20
	Lempira	0	10	10	0	10	10
	La Paz	0	10	0	0	10	0
	Ocotepeque	0	10	0	0	4	0
	Santa Bárbara	0	180	280	0	140	230
	Overall	0	60	60	0	50	50
Urban Areas (Market)	Copán	0	20	60	0	20	50
	Intibucá	0	20	30	0	10	30
	Lempira	0	0	20	0	0	10
	La Paz	0	30	60	0	30	50
	Ocotepeque	ND ^f^	ND	ND	ND	ND	ND
	Santa Bárbara	0	260	510	0	210	410
	Overall	0	70	100	0	60	80

^a^ Producer, samples collected from smallholder farmers in rural areas; Market, samples collected from retail stores in urban areas. ^b^ Women, daily intake of maize: 0.567 kg, body weight: 58.1 kg; Men, daily intake of maize: 0.60 kg, body weight: 76.2 kg. ^c^ Probable Daily Intake (PDI) based on the 10th percentile concentration of aflatoxins in the samples from the department. ^d^ Probable Daily Intake (PDI) based on the average concentration of aflatoxins in the samples from the department. ^e^ Probable Daily Intake (PDI) based on the 90th percentile concentration of aflatoxins in the samples from the department. ^f^ ND = Not Detected (below the limit of detection of the method, 10 ng/kg).

**Table 3:** Total fumonisin contamination levels in maize grain destined for human consumption and for animal feed in western Honduras.

Sample Source (type) ^a^	Department Name	Number of Samples						Prevalence (%) ^b^	Frequency Distribution (mg/kg)			
		Total	Concentration Range (mg/kg)						Mean ± SE ^c^	Median	10th Percentile	90th Percentile
			x < LOD	LOD ≤ x < 1	1 ≤ x < 3	3 ≤ x < 4	x ≥ 4					
Producer (Human consumption)	Copán	111	2	22	49	9	29	98	3.5 ± 0.4 ^z^	2.0	0.6	7.8
	Intibucá	80	3	26	32	3	16	96	2.6 ± 0.3 ^z^	1.6	0.5	5.9
	Lempira	103	3	32	39	11	18	97	2.3 ± 0.2 ^z^	1.7	0.5	5.2
	La Paz	64	3	21	25	6	9	95	2.1 ± 0.3 ^z^	1.4	0.4	5.0
	Ocotepeque	54	1	13	22	4	14	98	3.1 ± 0.4 ^z^	2.2	0.6	7.5
	Santa Bárbara	94	2	27	35	5	25	98	2.7 ± 0.2 ^z^	2.0	0.6	5.9
	Overall ^d^	506	14 (3%)	141 (28%)	202 (40%)	38 (7%)	111 (22%)	97 ^u^	$\bar{x}$ ^c^ = 2.7 ± 0.1 ^z^			
Market (Human consumption)	Copán	43	0	10	19	6	8	100	2.5 ± 0.3 ^z^	2.0	0.7	4.9
	Intibucá	16	0	2	6	1	7	100	3.5 ± 0.7 ^y,z^	3.1	0.8	7.1
	Lempira	17	2	3	5	2	5	88	3.0 ± 0.5 ^y,z^	2.6	0.6	5.3
	La Paz	14	0	1	4	0	9	100	5.4 ± 1.0 ^y^	5.3	1.9	8.0
	Ocotepeque	8	1	1	3	1	2	88	3.0 ± 0.9 ^y,z^	2.6	0.6	5.8
	Santa Bárbara	27	0	7	11	4	5	100	2.9 ± 0.5 ^z^	2.3	0.8	5.3
	Overall	125	3 (2%)	24 (19%)	48 (39%)	14 (11%)	36 (29%)	98 ^u^	$\bar{x}$ = 3.2 ± 0.2 ^z^			
Producer (Animal feed)	Copán	30	0	0	4	3	23	100	9.9 ± 1.0 ^y^	11.0	2.6	15
	Intibucá	6	0	1	2	0	3	100	3.5 ± 1.1 ^y,z^	3.1	1.1	6.4
	Lempira	27	1	1	4	4	17	96	9.7 ± 1.4 ^y^	12.0	1.8	15
	La Paz	11	1	4	2	1	3	91	3.5 ± 1.3 ^z^	1.1	0.3	11
	Ocotepeque	6	1	0	2	0	3	83	5.3 ± 2.3 ^y,z^	3.2	0.7	12
	Santa Bárbara	29	1	4	12	1	11	97	5.9 ± 1.1 ^y,z^	2.1	0.9	14
	Overall	109	4 (4%)	10 (9%)	26 (24%)	9 (8%)	60 (55%)	96 ^u^	$\bar{x}$ = 7.6 ± 0.6 ^y^			

^a^ Producer, samples collected from smallholder farmers in rural areas; Market, samples collected from retail stores in urban areas. ^b^ Prevalence was defined as the number of samples with fumonisin concentration exceeding the limit of detection (LOD) of 0.25 mg/kg. ^c^ Average concentration of fumonisin in total samples ± standard error. Average fumonisin values among departments, within the same sample source, that share the same superscript letter (y, z) are not significantly different from one another (*p* > 0.05). ^d^ Overall prevalence (u) and concentration (x, y, z) values with the same superscript letter do not differ significantly (*p* > 0.05).

**Table 4:** Dietary exposure to fumonisins in maize grain consumed by households in rural and urban areas of western Honduras.

Sample Source ^a^	Department Name	Women (µg/kg bw/day) ^b^			Men (µg/kg bw/day) ^b^		
		10th Percentile ^c^	Average ^d^	90th Percentile ^e^	10th Percentile ^c^	Average ^d^	90th Percentile ^e^
Rural Areas (Producer)	Copán	6.2	34	76	5.0	27	61
	Intibucá	5.2	25	58	4.2	20	47
	Lempira	4.5	23	51	3.7	18	41
	La Paz	4.0	21	49	3.2	17	39
	Ocotepeque	5.9	30	73	4.8	24	59
	Santa Bárbara	5.5	26	58	4.5	21	47
	Overall	5.1	27	57	4.1	22	46
Urban Areas (Market)	Copán	6.5	25	48	5.2	20	38
	Intibucá	7.9	34	69	6.4	28	56
	Lempira	5.7	29	52	4.6	24	42
	La Paz	19	53	78	15.1	43	63
	Ocotepeque	6.2	30	56	5.0	24	45
	Santa Bárbara	7.4	28	52	6.0	23	42
	Overall	6.6	31	64	5.3	25	52

^a^ Producer, samples collected from smallholder farmers in rural areas; Market, samples collected from retail stores in urban areas. ^b^ Women, daily intake of maize: 0.567 kg, body weight: 58.1 kg; Men, daily intake of maize: 0.60 kg, body weight: 76.2 kg. ^c^ Probable Daily Intake (PDI) based on the 10th percentile concentration of fumonisins in the samples from the department. ^d^ Probable Daily Intake (PDI) based on the average concentration of fumonisins in the samples from the department. ^e^ Probable Daily Intake (PDI) based on the 90th percentile concentration of fumonisins in the samples from the department.

## Data Availability

The data on which this study is based are openly available in K-State Research Exchange (K-REx) at https://hdl.handle.net/2097/42520 (accessed on 14 August 2023).
